# Isomorphic Fluorescent Nucleosides

**DOI:** 10.1021/acs.accounts.4c00042

**Published:** 2024-04-13

**Authors:** Yitzhak Tor

**Affiliations:** Department of Chemistry and Biochemistry, University of California, San Diego, 9500 Gilman Drive, La Jolla, California 92093, United States

## Abstract

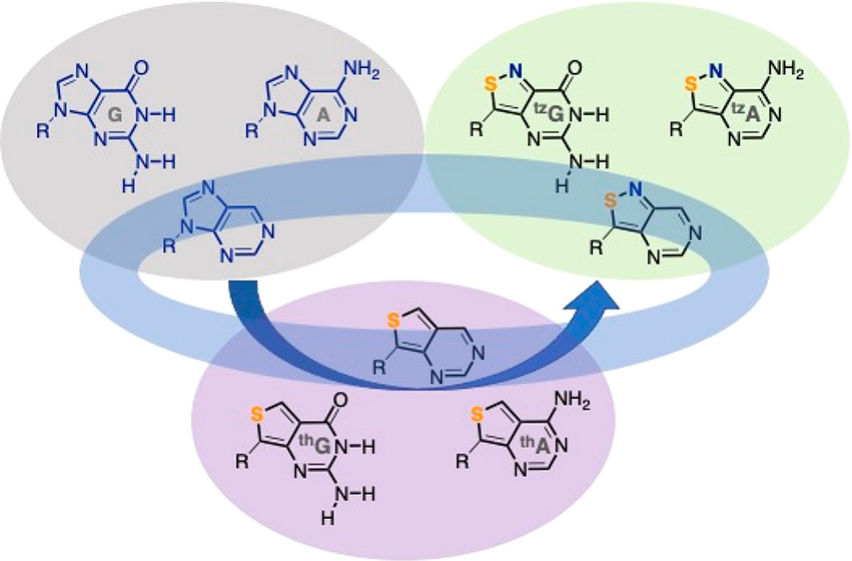

In 1960, Weber prophesied that “There are many ways in which the properties of the excited state can be utilized to study points of ignorance of the structure and function of proteins”. This has been realized, illustrating that an intrinsic and highly responsive fluorophore such as tryptophan can alter the course of an entire scientific discipline. But what about RNA and DNA? Adapting Weber’s protein photophysics prophecy to nucleic acids requires the development of intrinsically emissive nucleoside surrogates as, unlike Trp, the canonical nucleobases display unusually low emission quantum yields, which render nucleosides, nucleotides, and oligonucleotides practically dark for most fluorescence-based applications.

Over the past decades, we have developed emissive nucleoside surrogates that facilitate the monitoring of nucleoside-, nucleotide-, and nucleic acid-based transformations at a nucleobase resolution in real time. The premise underlying our approach is the identification of minimal atomic/structural perturbations that endow the synthetic analogs with favorable photophysical features while maintaining native conformations and pairing. As illuminating probes, the photophysical parameters of such isomorphic nucleosides display sensitivity to microenvironmental factors. Responsive isomorphic analogs that function similarly to their native counterparts in biochemical contexts are defined as isofunctional.

Early analogs included pyrimidines substituted with five-membered aromatic heterocycles at their 5 position and have been used to assess the polarity of the major groove in duplexes. Polarized quinazolines have proven useful in assembling FRET pairs with established fluorophores and have been used to study RNA–protein and RNA–small-molecule binding. Completing a fluorescent ribonucleoside alphabet, composed of visibly emissive purine (^th^A, ^th^G) and pyrimidine (^th^U, ^th^C) analogs, all derived from thieno[3,4-*d*]pyrimidine as the heterocyclic nucleus, was a major breakthrough. To further augment functionality, a second-generation emissive RNA alphabet based on an isothiazolo[4,3-*d*]pyrimidine core (^th^A, ^tz^G, ^tz^U, and ^tz^C) was fabricated. This single-atom “mutagenesis” restored the basic/coordinating nitrogen corresponding to N7 in the purine skeleton and elevated biological recognition.

The isomorphic emissive nucleosides and nucleotides, particularly the purine analogs, serve as substrates for diverse enzymes. Beyond polymerases, we have challenged the emissive analogs with metabolic and catabolic enzymes, opening optical windows into the biochemistry of nucleosides and nucleotides as metabolites as well as coenzymes and second messengers. Real-time fluorescence-based assays for adenosine deaminase, guanine deaminase, and cytidine deaminase have been fabricated and used for inhibitor discovery. Emissive cofactors (e.g., S^th^AM), coenzymes (e.g., N^tz^AD^+^), and second messengers (e.g., c-di-^tz^GMP) have been enzymatically synthesized, using ^xy^NTPs and native enzymes. Both their biosynthesis and their transformations can be fluorescently monitored in real time.

Highly isomorphic and isofunctional emissive surrogates can therefore be fabricated and judiciously implemented. Beyond their utility, side-by-side comparison to established analogs, particularly to 2-aminopurine, the workhorse of nucleic acid biophysics over 5 decades, has proven prudent as they refined the scope and limitations of both the new analogs and their predecessors. Challenges, however, remain. Associated with such small heterocycles are relatively short emission wavelengths and limited brightness. Recent advances in multiphoton spectroscopy and further structural modifications have shown promise for overcoming such barriers.

## Key References

- ShinD.; SinkeldamR. W.; TorY.Emissive RNA Alphabet. J. Am. Chem. Soc.2011, 133, 14912–1491510.1021/ja206095a21866967 PMC3179766.^1^ A fluorescent ribonucleoside alphabet, composed of emissive purine (^th^A, ^th^G) and pyrimidine (^th^U, ^th^C) analogs, is derived from thieno[3,4-*d*]pyrimidine as the heterocyclic nucleus. They display highly desirable traits, including native Watson–Crick faces, unparalleled structural isomorphicity, and visible emission.
- SinkeldamR. W.; McCoyL. S.; ShinD.; TorY.Enzymatic Interconversion of Isomorphic Fluorescent Nucleosides: Adenosine Deaminase Transforms an Adenosine Analogue into an Inosine Analogue. Angew. Chem., Int. Ed.2013, 52, 14026–1403010.1002/anie.201307064PMC394749724288262.^2^^th^A serves as an emissive substrate for adenosine deaminase. The enzymatic deamination yields the distinctly emissive inosine analogs ^th^I, facilitating real-time monitoring of the enzyme-catalyzed reaction and its inhibition.
- RoviraA.; FinA.; TorY.Chemical mutagenesis of an emissive RNA alphabet. J. Am. Chem. Soc.2015, 137, 14602–1460510.1021/jacs.5b1042026523462 PMC5281058.^3^ To augment isomorphicity and functionality, a second-generation fluorescent ribonucleoside alphabet, based on isothiazolo[4,3-*d*]pyrimidine, was constructed (including ^tz^A, ^tz^G, ^tz^U, and ^tz^C). This single-atom “mutagenesis” restores the basic/coordinating nitrogen corresponding to N7 in the purine skeleton.
- HadidiK.; SteinbuchK. B.; DozierL. E.; PatrickG. N.; TorY.Inherently Emissive Puromycin Analogues for Live Cell Labelling. Angew. Chem., Int. Ed.2023, 62, e20221678410.1002/anie.202216784PMC1021313936973168.^4^ Puromycin analogs containing a ^th^A core, modified with substituted azetidines, inhibit translation and generate emissive translation products without any follow-up chemistry. The 3,3-difluoroazetidine-containing derivative can be visualized in both live and fixed HEK293T cells and rat hippocampal neurons.

## 1 Introduction

Fluorescence-based tools have revolutionized modern science. Advances in instrumentation and techniques have broadened this phenomenon’s outreach from fundamental spectroscopic explorations to incredible imaging tools. The emergence of powerful light sources, miniaturization, and elevated computer power has driven impressive accomplishments, yet all techniques and applications fundamentally rely on suitable chromophores. It is clear how fluorescent proteins have altered the landscape of biology.^5^ Less is apparent about the evolution, features, and constraints of low-molecular-weight fluorophores, particularly in the field of nucleic acid biophysics.^6,7^

**Figure 1:**
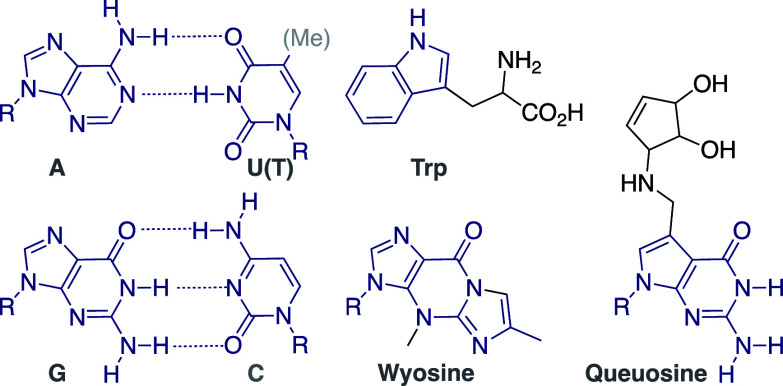
Canonical Watson–Crick pairs (left) and examples of naturally occurring intrinsic fluorophores (e.g., tryptophan, Trp). For reference, the absorption and emission maxima of Trp are ca. 280 and 350 nm, respectively, and Wyosine’s are 318 and ca. 450 nm, respectively. (Note that these values are susceptible to microenvironmental polarity.^6,10^) R = d-ribose or 2′-deoxy-d-ribose.

The photophysics of RNA and DNA is unique among biomolecules that possess chromophoric components. Unlike proteins, which contain intrinsically fluorescent amino acids (e.g., Trp), the canonical nucleosides, which display absorption maxima between 250 and 270 nm, are practically nonemissive.^8,9^ With the exception of a few rare, emissive modified nucleosides (e.g., wyosine), the predominant purines (adenine and guanine) and pyrimidines (uracil/thymine and cytosine) and their corresponding nucleosides (A, G, U/T, and C, respectively) display unique excited-state dynamics, associated with effective nonradiative decay pathways (Figure 1).^8^ This results in exceptionally short excited-state lifetimes (τ = 0.2–0.7 ps) when compared to common organic fluorophores (τ = 0.5–20 ns) and hence extremely low fluorescence quantum yields under neutral conditions (ϕ_F_ = 0.5 × 10^–4^–3 × 10^–4^). These critical building blocks are thus effectively “dark” for most standard applications.^8−10^

A common workaround, especially for oligonucleotides, involves appending established fluorophores to their termini or to the nucleobases.^11^ Modern coupling and biorthogonal click reactions have popularized such approaches for both in vitro and in vivo labeling. End or edge labeling is, however, far from optimal: (a) such labels are typically insensitive to remote binding events and do not provide “nucleoside-specific” information; (b) such fluorophores are typically large, charged, and frequently noninnocent.^12^ Furtheremore, such approaches are not suitable for tagging low-molecular-weight nucleotide-based cofactors and messengers, as the fluorophores might be as large as the tagged bioactive core itself.

The potential impact of intrinsically fluorescent nonperturbing nucleosides can be inferred from Gregorio Weber’s prophecy. In a 1960 conference on “Light and Life” he stated the following: “There are many ways in which the properties of the excited state can be utilized to study points of ignorance of the structure and function of proteins”.^13^ Following Weber’s seminal work on the fundamental photophysics of fluorescent amino acids,^14^ hundreds of papers, where Trp residues (either native or engineered) are exploited to study the structure, activity, and recognition of proteins, appear every year. Can a parallel methodology be advanced for nucleic acids, where a single nucleotide is replaced with a faithful emissive surrogate, thus opening a nonperturbing optical window into global and local features of such biomolecules?

Since the early 2000s, we have focused on the design, synthesis, and implementation of minimally perturbing and responsive fluorescent nucleosides, aiming to advance effective probes to monitor nucleoside-, nucleotide-, and nucleic acid-based transformations at a nucleobase “resolution” in real time. The ambition was to “trick” biology into seamlessly accepting such analogs as faithful surrogates of endogenous substrates, while their alternate photophysics would open a window into processes that were otherwise undetectable by fluorescence spectroscopy. Understandably, perturbations are inevitable when replacing native residues with a synthetic analog, even if minimally altered. We defined nucleosides that faithfully mimic the size, shape, hybridization and recognition features of the native nucleobases as isomorphic. They should ideally possess red-shifted absorption bands to minimize spectral overlap with the natural bases and display visible emission and adequate emission quantum yields. To serve as reporters, the analogue’s photophysical parameters (λ_em_ and/or ϕ_F_, τ) must relay microenvironmental changes, thus being responsive. Analogs performing closely to their native counterparts in numerous contexts are classified as isofunctional.

Notably, idealized nucleoside analogs can be envisaged and frequently synthesized, but their ultimate photophysical features are not a “designer property”. Readily available computational tools have a limited guiding ability when it comes to excited-state dynamics. Furthermore, the energetic and dynamic susceptibility of excited states to environmental factors (polarity, viscosity, etc.) and to inter- and intramolecular quenching pathways further limits the predictability of a chromophore’s photophysical behavior in diverse contexts. Fabricating new and effective fluorophores thus remains an empirical and iterative process. This impacts the discovery cycle of fluorescent nucleosides: engaging in lengthy “total syntheses” of complex heterocycles prior to obtaining an indication of potentially useful photophysical features is impractical. Furthermore, heterocycles that deviate from the canonical pyrimidines and purines frequently populate multiple tautomeric forms, which could complicate their implementation. A common practice, which does not circumvent the problem entirely,^15^ is to synthesize and evaluate the nucleobase prior to fabricating the corresponding nucleoside(s). A systematic assessment of the photophysical features and their dependence on polarity (ideally studied in solvent mixtures such as water/dioxane and not in pure solvents) and correlations with microscopic solvent polarity scales, such as Reichardt’s E_T_(30), is crucial.^10,16^

## 2 Inspiration and Early Explorations

**Figure 2:**
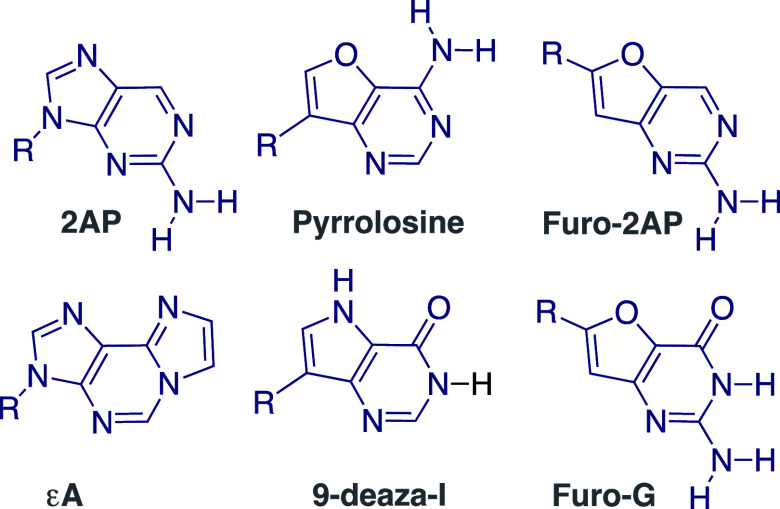
Synthetic and naturally occurring nucleosides serve as inspiration.

The archetypal and inspirational and perhaps the standard to which every new fluorescent nucleoside has been compared is 2-aminopurine (2AP) riboside, which was introduced along with formycin and 2,6-diaminopurine riboside in 1969 by Lubert Stryer (Figure 2).^17^ As a constitutional isomer of adenine, 2AP strikingly illustrates how a simple molecular change can radically alter a chromophore’s photophysics. Despite being perturbing,^18^ 2AP has served as a biophysical workhorse for over five decades.^19^ With excitation and emission around 303 and 370 nm, respectively, and quantum yield of 0.68 (in water), 2AP-riboside has found enormous utility, exploiting its exquisite responsiveness, as demonstrated by us^20^ and many others.^19^ This environmental susceptibility is also its Achilles’ heel. 2AP is considerably quenched in single- and double-stranded constructs. Defining the other boundary and triggering reserved optimism was Nelson Leonard’s εA (Figure 2).^21,22^ With its extended footprint and blocked Watson–Crick face, ethenoA would be considered to be highly perturbing, yet numerous applications have nevertheless demonstrated how useful an emissive nucleoside, even disturbing, could be when strategically implemented.^22^

Our early explorations had been guided by the photophysical characteristics of 5,6-fused heterocycles (e.g., benzofurans and benzothiophenes), which display red-shifted absorption and emission maxima and drastic fluorescence enhancement compared to their constituents or to imidazolo-based heterocycles. The absorption maximum of pyrrolosine (a naturally occurring C-nucleoside that was incorrectly thought to be the isomeric 9-deazainosine^23^), for example, is red-shifted by 15 nm compared to that of adenosine (Figure 2). Susan Seaman produced model furanopyrimidines (Figure 2), which indeed displayed red-shifted absorption and emission maxima. While the trivially named Furu-G and Furu-2AP were modestly emissive (ϕ_F_ < 0.1), their emission maxima remained below 390 nm. Coupled to their lengthy synthesis, they were deemed suboptimal at that stage of our nascent program.

## 3 Conjugated Emissive Nucleosides

**Figure 3:**
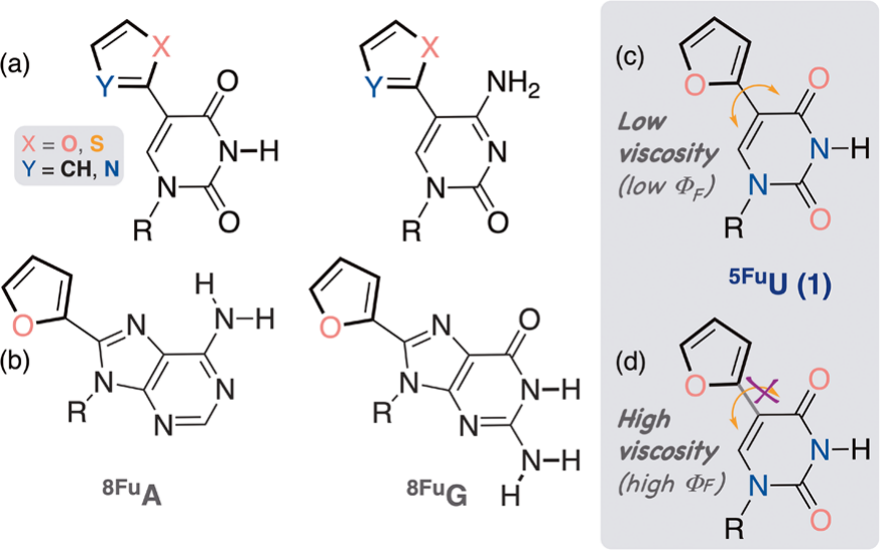
5-Modified pyrimidines (a) and 8-modified purines (b). As molecular rotors, fluorescence intensity responds to viscosity and temperature changes (c, d).^26^ Note, both ribonucleosides and 2′-deoxy-ribonucleosides can be made.

Pyrimidines, substituted at their 5-position with five-membered aromatic heterocycles, were prepared by Nick Greco, utilizing palladium-mediated cross coupling reactions (Figure 3a).^24^ Crystal structures showed the same solid-state conformational preferences as thymidine, with anti and 2′-endo conformations for the heterocycle and 2′-deoxy-d-ribose, respectively.^25^ They emit in the visible range (maxima at 390–443 nm, decaying >500 nm) and have large Stokes shifts (8400–9700 cm^–1^).^24,25^ Acting as molecular rotors, where rotation around the biaryl bond provides an effective channel for nonradiative torsional relaxation (Figure 3c), they have low emission quantum yields in nonviscous media (ϕ_F_ = 0.01–0.035), which dramatically increases with elevated viscosity (Figure 3d).^26^ These fluorophores thus exhibit sensitivity to both microenvironmental polarity and crowding effects. While the rotor element unifies all, endowing high sensitivity to viscosity, the distinct electronic relationship between the pyrimidine core and the appended five-membered heterocycle dictates unique excited-state manifolds and CT characteristics and hence diverse Stokes shifts and solvatochromic behavior.^26^

These 5-aryl pyrimidines can be incorporated into oligonucleotides by either solid-phase or enzymatic synthesis, causing no destabilization of hybridized double-stranded constructs.^27^ Despite the apparent “primitive” design, this motif has been exploited in assays, as demonstrated by Nicholas Greco^28^ and Seergazhi Srivatsan,^27^ and its utility has later been expanded by Seergazhi Srivatsan and Richard Manderville.^29,30^ The linear correlation between the Stokes’ shift and microenvironmental polarity of ^5Fu^dU (**1**, Figure 3c,d) was used to assess the polarity of major grooves in DNA and DNA/RNA duplexes.^31^ A significant emission enhancement was also seen for ^5Fu^dU when placed opposite an abasic site, compared to a perfect match, and was rationalized by its helix internalization and limited rotational flexibility.^26^

**Figure 4:**
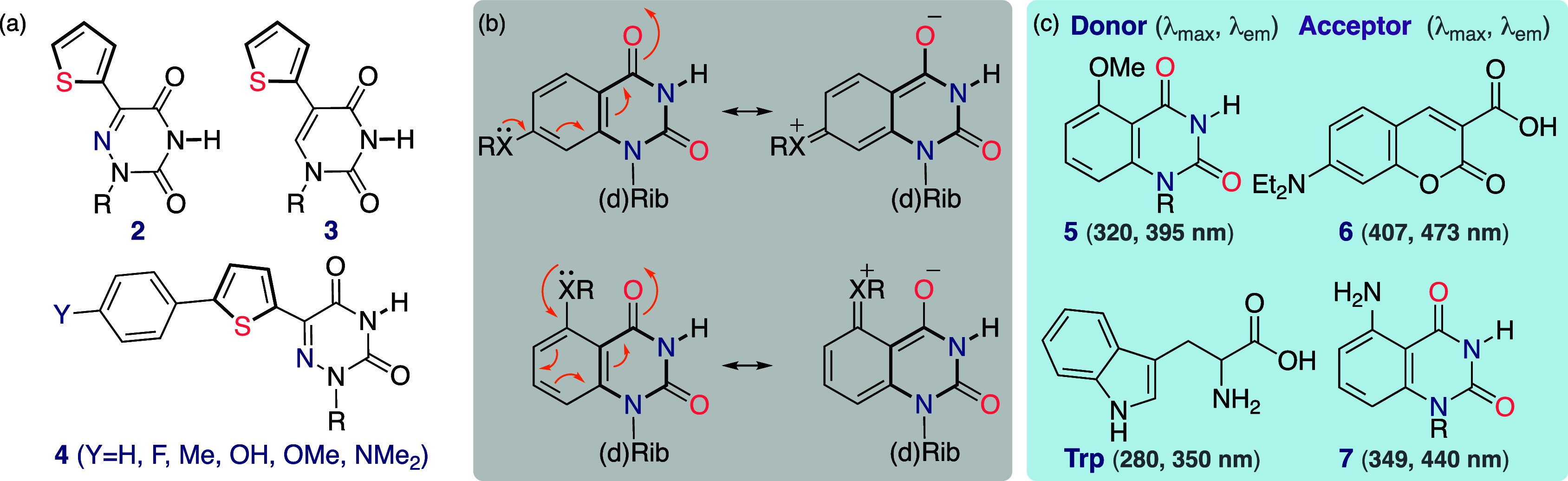
(a) Emissive aza-pyrimidines (e.g., **2**, **4**) compared to the “parent” extended pyrimidine (**3**), (b) polarized quinazolines as expanded pyrimidines, and (c) experimentally established FRET pairs.

The 8-furano-modified adenosine and guanosine derivatives (Figure 3b) are very emissive (ϕ_F_ = 0.69 and 0.57, respectively) and responsive to both polarity and temperature.^25^ Since their emission peaked below 380 nm and 8-substituted purines are frequently perturbing due to their syn conformational preference, we abandoned these emitters. Renatus Sinkeldam and Patrycja Hopkins discovered that replacing the pyrimidine core with the corresponding 1,2,4-triazine yields a bathochromic shift, a hyperchromic effect, and higher brightness (e.g., **2**, Figure 4a).^32^ Compared to **3**, **2** displays a red-shifted emission (λ_em_= 434 and 455 nm, respectively) and a considerably higher quantum yield (ϕ_F[H_2_O]_ = 2 and 20%, respectively). By extending this core and enhancing the charge-transfer character, visibly emitting analogs **4** were obtained (λ_em_ 490–575 nm, ϕ_F(H_2_O)_ = 0.02–0.24).^33^ Their Stokes shifts were found to correlate with Hammett σ_*p*_ and σ_*p*_^*+*^ parameters.^10^

As relatively small chromophores with limited charge transfer character in the ground state, the 5-extended pyrimidines and 8-modified purines (Figure 3) absorb around the high-energy end of the visible spectrum. Such photophysical features would typically limit their biological and imaging practicality. It was therefore prudent to explore their multiphoton-induced emission. In collaboration with Steven Magennis, it was found that thiophene-containing pyrimidines **2** and **3** display unusually high cross sections for two-photon excitation (3.8 and 7.6 GM, respectively, excitation at 690 nm), far exceeding other emissive nucleosides such as 2AP.^34^ The highly polarized **4** (Y = NMe_2_, Figure 4) displayed single-molecule brightness over an order of magnitude higher than for any fluorescent base analog, at the time, under both 2P and 3P excitation, facilitating the first single-molecule detection of an emissive nucleoside with multiphoton excitation, by Steven Magennis and Anita Jones.^35^

## 4 FRET Pairing

To FRET pair emissive nucleosides with common fluorophores, Yun Xie prepared polarized quinazolines as expanded pyrimidines (Figure 4b). While deviating from the strictest definition of isomorphicity, this heterocycle provided tunable photophysical control via substitution at the conjugated 5 and 7 positions, yielding a wide emission range (350–500 nm). 5-Methoxyquinazoline-2,4-(1*H*,3*H*)-dione (**5**) is an ideal donor for 7-diethylaminocoumarin-3-carboxylic acid derivatives (**6**, Figure 4c) and was used for a discovery assay for antibiotics targeting the bacterial ribosomal A-site (e.g., aminoglycosides).^36^ By judiciously selecting an additional FRET pair, assembling a three chromophore system, an assay for assessing the selectivity of aminoglycoside-related antibiotics for prokaryotic vs eukaryotic decoding sites was fabricated.^37^ Intriguingly, the 5-amino derivative (**7**) is a FRET acceptor for Trp’s indole (Figure 4c). This facilitates the monitoring of RNA–peptide/protein interactions by relying on native, intrinsically fluorescent Trp residues,^38^ which are disproportionally abundant within RNA binding domains.^39,40^ The potential of this FRET pair has been demonstrated by studying the association of the HIV-1 Rev peptide with the Rev response element (RRE), its endogenous RNA target.^38^

## 5 Emissive RNA Alphabets

**Figure 5:**
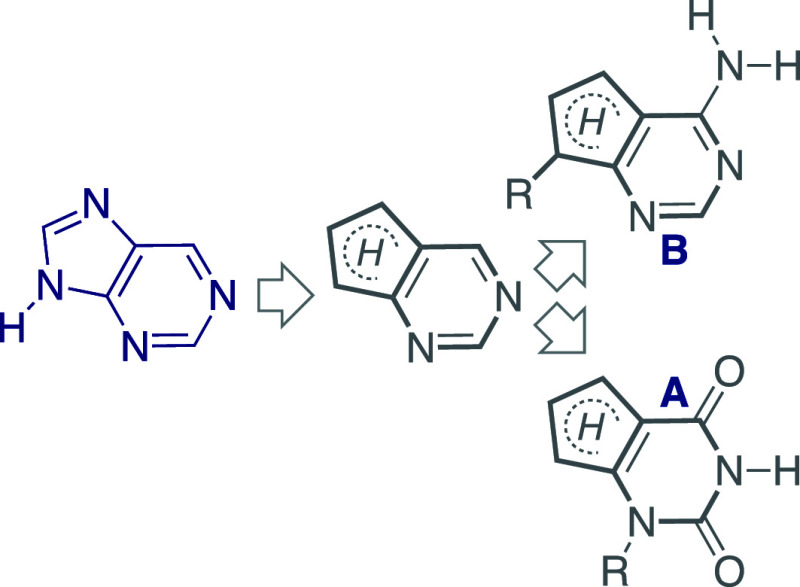
General design of emissive purines (top) and pyrimidines (bottom), derived from a single core heterocycle.

Conceptually, one can view fused pyrimidines as fundamental heterocycles for both emissive purines and pyrimidines (Figure 5). As a generic heterocyclic platform, *N*-glycosylation could provide emissive pyrimidine analogs (**A**), while *C*-glycosidation at the 5-membered ring would yield fluorescent purine analogs (**B**), assuming the central motif displays favorable photophysics.^41^ Functional group elaboration can expand these heterocycles into isosteric Watson–Crick-like alphabets.

**Figure 6:**
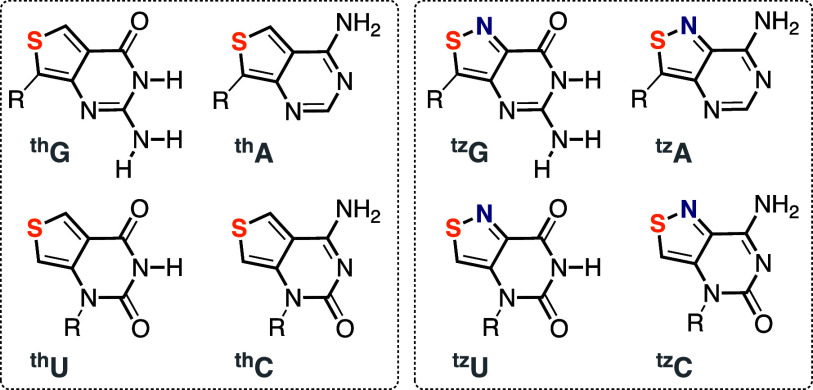
First generation (left, i.e., thiophenopyrimidine-based^1^) and second generation (right, i.e., isothiazolopyrimidine-based^3^) emissive nucleoside alphabets.

**Figure 7:**
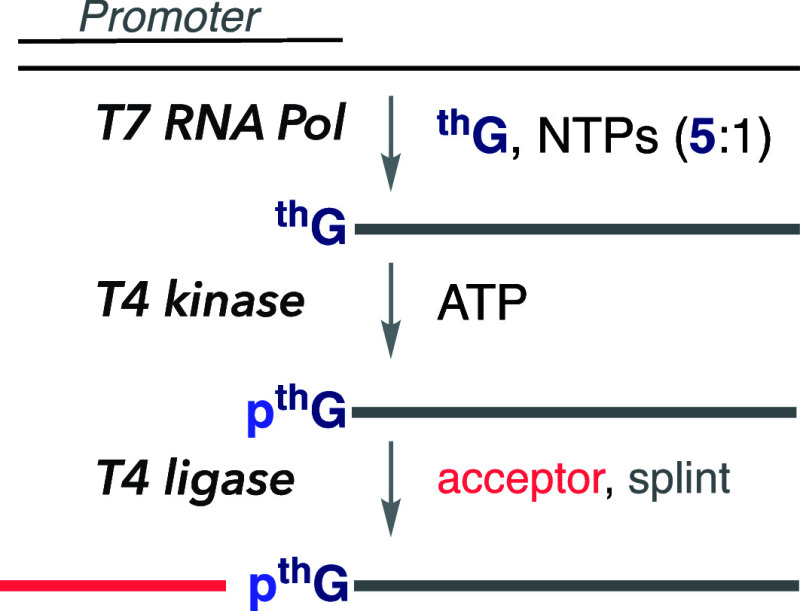
Enzymatic approach to singly labeled RNA constructs via ^th^G-enforced transcription initiation.^43^

In 2011, Dongwon Shin had completed a fluorescent ribonucleoside alphabet, composed of highly emissive purines (^th^A, ^th^G) and pyrimidines (^th^U, ^th^C), all (philosophically) derived from thieno[3,4-*d*]pyrimidine as the heterocyclic nucleus (Figure 6).^1^ The structural, biophysical, and spectroscopic characteristics of this ribonucleoside set show desirable traits, including unparalleled structural isomorphicity, minimal perturbation upon incorporation into duplexes, and relatively intense visible emission.^1^ Lisa McCoy showed that ^th^GTP is seamlessly accepted by T7 RNA polymerase as a GTP surrogate *in vitro*.^42^^th^GTP thus initiates transcription reactions and elongates the nascent transcripts, yielding bright per-modified RNA oligonucleotides. To enzymatically fabricate site-specifically modified RNAs, Yao Li demonstrated that transcription can be initiated with excess ^th^G (plus native NTPs).^43^ The resultant 5′-^th^G-terminated transcript is phosphorylated and ligated, yielding a singly labeled emissive RNA construct (Figure 7).^43^ To demonstrate the utility of this protocol, several altered hammerhead (HH) ribozymes and a singly modified HH substrate were fabricated. By strategically modifying key positions, mechanistic insight into the ribozyme-mediated cleavage was gained. Additionally, the emissive features of the modified nucleoside and its responsiveness to environmental changes have been used to monitor cleavage in real time by steady-state fluorescence spectroscopy.^43^ Notably, Venkat Gopalan disclosed a refined methodology where a mutant T7 RNA polymerase has shown an improved ability to accommodate our emissive guanosine surrogate.^44^ It is worthwhile to note that such protocols can be expanded into other emissive purine analogs.

This emissive alphabet has been subjected to unsolicited theoretical analyses, correlating our experimental observations with calculated values.^45^ Experimentally, the ribo- and deoxynucleosides have been applied for detecting depurination of rRNA’s α-sarcin/ricin loop by RIPs (e.g., ricin),^46^ as building blocks for emissive siRNA,^47^ for monitoring B–Z transitions,^48^ for PCR labeling of long DNA,^49^ for studying the UHRF1-mediated flipping dynamics of methylated cytosines,^50^ for monitoring translation,^51^ for assessing codon stringency,^52^ and for studying nucleosome structures.^53^ Yves Mély’s studies have been particularly noteworthy, assessing ^th^G in diverse environments using steady-state, time-resolved, and anisotropy measurements, which were complemented by theoretical calculations and a side-by-side comparison to 2AP.^54^ 2AP’s emission is quenched in single- and particularly double-stranded oligonucleotides, and the associated fluorescence intensity decays are commonly complex, displaying multiple lifetimes. Critically, species with extremely short excited-state lifetimes (i.e., “dark” species), while frequently reflecting biologically relevant folds, are overshadowed by detectable emissive states that might be associated with biologically irrelevant conformations. These complications are resolved, by and large, by ^th^G.^55^ Fabricating highly emissive purine analogs, thus “relaxing” the community’s dependence on 2AP as a “universal” emissive nucleoside, was the most rewarding aspect of this first-generation alphabet.

**Figure 8:**
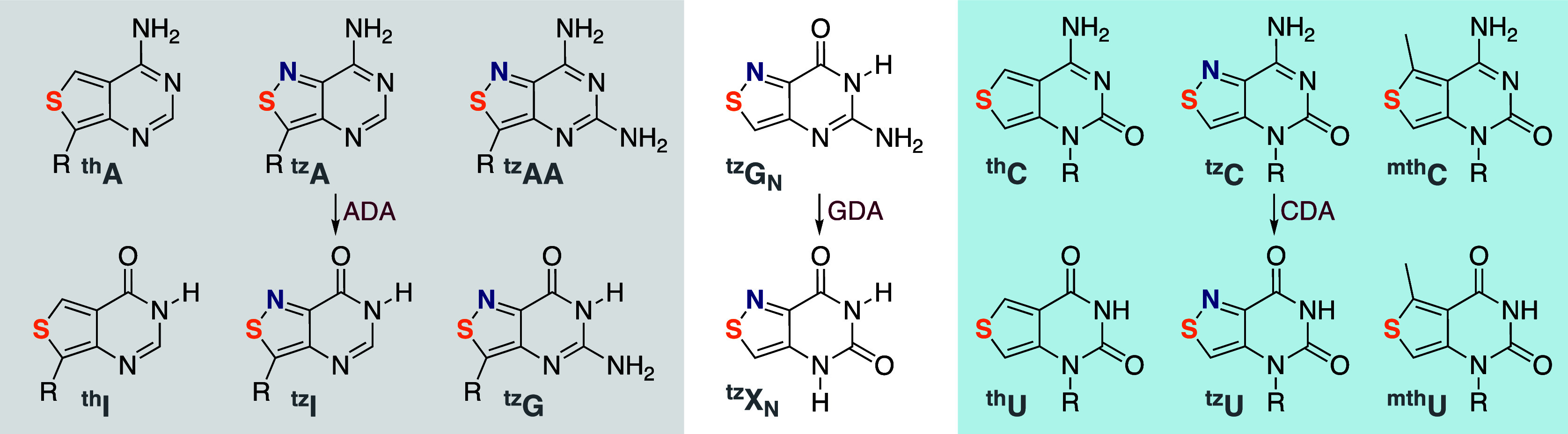
Emissive nucleosides and nucleobases can be deaminated by native enzymes. Adenosine deaminase (ADA) deaminates ^tz^A as effectively as adenosine, but ^th^A, lacking the basic nitrogen at the position corresponding to the purines’ N7, is a poorer substrate. Similarly, human GDA does not deaminate ^th^G_N_, the corresponding thiophenoguanine nucleobase. In contrast, CDA displays promiscuity and deaminates even perturbing analogs, such as ^mth^C.

Deaminases were adopted as demanding “functionality litmus tests”. Renatus Sinkeldam showed that adenosine deaminase (ADA1), a key metabolic enzyme, deaminates ^th^A, yielding the corresponding distinctly emissive inosine analog ^th^I (Figure 8).^2^ This allows one to monitor the enzyme-catalyzed reaction and its inhibition in real time. Michaelis–Menten kinetic analyses showed, however, a 15-fold higher *K*_M_ for ^th^A compared to that of adenosine.^2^ This was attributed to the missing basic nitrogen in the former, corresponding to N7 in the purine skeleton. To restore the basic/coordinating nitrogen and augment the isomorphicity and functionality, a second-generation emissive RNA alphabet, based on isothiazolo[4,3-*d*]pyrimidine, was constructed by Alex Rovira (Figure 6).^3,56^ Real-time measurements show that ADA deaminates ^tz^A to ^tz^I at the same rate as it does A to I.^3^

The preparation of multiple emissive ADA substrates (Figure 8), displaying distinct MM kinetics and photophysical parameters, facilitated the fabrication of a high-throughput assay for discovering inhibitors for this zinc enzyme.^57^ In collaboration with Seth Cohen’s laboratory, Paul Ludford had screened >300 metal-binding pharmacophores, identifying novel inhibitory motifs.^58^ In addition to metabolic A to I deamination, mRNA editing and tRNA maturation via adenosine deaminases acting on RNAs (ADARs) and adenosine deaminases acting on tRNAs (ADATs) involve similar transformations in distinct oligomeric contexts. With Peter Beal, we evaluated the reactivity of ADAR2 with the ^th^A-modified GluR B mRNA R/G editing site. The RNA was deaminated rapidly to yield the ^th^I-containing product strand (*k*_rel_^th^A/A = 2.1), and the process could be monitored by changes in the fluorescence of the modified RNA.^59^ The higher deamination rate by ADAR2 vs ADA is likely due to the insensitivity of the former to N7 modifications.^60^

The applicability of other emissive nucleobases and nucleosides as potential substrates for metabolic enzymes was also evaluated with guanine deaminase (GDA) and cytidine deaminase (CDA), two hydrolytic zinc-based enzymes (Figure 8). Marcela Bucardo showed that the emissive isothiazolo-guanine analog ^tz^G_N_ is an excellent substrate for human GDA (while ^th^G_N_ is not), facilitating real-time monitoring of deamination and its inhibition.^61^ Paul Ludford showed that ^th^C, ^tz^C, and even ^mth^C were viable fluorescent substrates for CDA (Figure 8).^62^

## 6 Emissive Cofactors and Messengers

**Figure 9:**
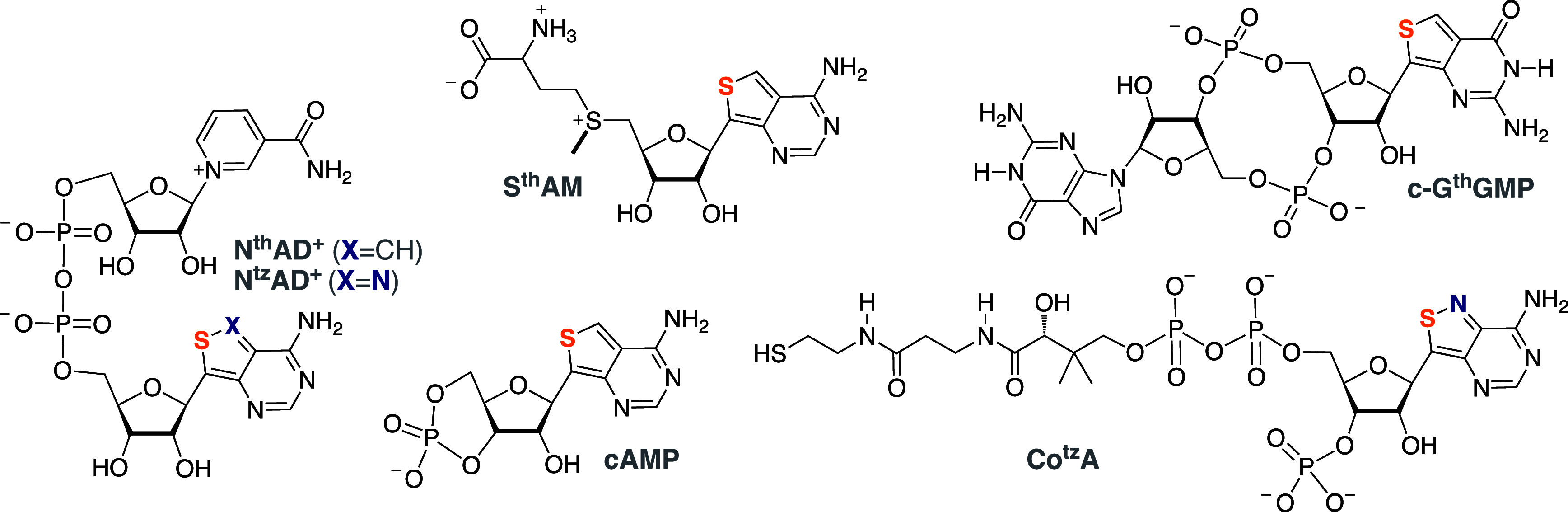
Nucleoside cofactors/messengers that can be synthesized enzymatically.

**Figure 10:**
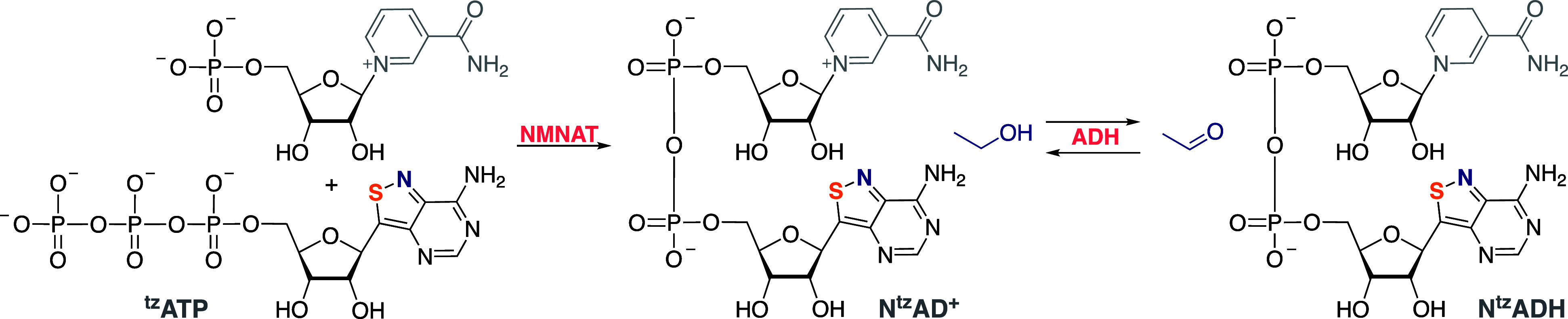
Enzymatic synthesis of N^tz^AD^+^ and engagement in redox reactions with alcohol dehydrogenase.

Most nucleotide-based cofactors and second messengers are devoid of any distinguishable fluorescence. Early studies, pioneered by Nelson Leonard^22^ and David Shugar,^63^ used perturbing emissive analogs (e.g., εA) or weakly emissive ones (e.g., 8-azapurines). As these cofactors typically contain adenosine as the nucleoside/tide moiety, the isomorphic emissive purine analogs developed in our laboratory appeared to be potentially suitable for congener fabrication (Figure 9). Early on, Charlotte Vranken employed SalL, an enzyme that catalyzes the synthesis of SAM from 5′-chloro-5′-deoxyoadenosine and l-methionine, to prepare S^th^AM using the corresponding chlorinated ^th^A as a substrate.^64^ The resulting SAM analog was shown to replace the native cofactor in DNA methylation reactions.^64^ Alex Rovira chemically synthesized the emissive N^tz^AD^+^ (Figure 9) and showed that its enzymatic reduction in the presence of ethanol and alcohol dehydrogenase, yielding the corresponding N^tz^ADH, is associated with significant fluorescence quenching.^65^ This reflects a “mirror image” and a complementary photophysical response to native NAD^+^/NADH that has been extensively employed in “classical” biochemical assays (relying on the inherent fluorescence of NADH, the reduced form). Francois Hallé and Andrea Fin illustrated that N^tz^AD^+^ and N^tz^ADP^+^ can also be enzymatically synthesized using ^tz^ATP and native enzymes (Figure 10).^66^ Thus, NMNAT condenses ^tz^ATP and nicotinamide mononucleotide to yield N^tz^AD^+^, which can seamlessly engage in NAD^+^-based transformations. Treating N^tz^AD^+^ with NAD kinase and ATP (or ^tz^ATP) produces the 2′-phosphorylated N^tz^ADP^+^, which can partake in NADP-specific enzymatic transformations.^66^ While phosphorylation is expectedly photophysically “silent”, all redox-based reactions are associated with significant fluorescence changes that facilitate their real-time monitoring.

Isomorphic emissive NAD^+^ analogs can serve as substrates for other NAD^+^-consuming enzymes, including PARPs. Jonas Feldmann showed that N^tz^AD^+^ also serves as a substrate for ribosyl transferases, including human adenosine ribosyl transferase 5 and cholera toxin subunit A, which hydrolyze the nicotinamide and transfer ^tz^ADP-ribose to an Arg analog, respectively.^67^ These reactions could be monitored by fluorescence spectroscopy, in stark contrast to the corresponding processes with the native and nonemissive NAD^+^.^66^ The “N7-lacking” N^th^AD^+^ showed reduced compatibility relative to that of N^tz^AD^+^ (Figure 9). The distinct tolerance, displayed by diverse NAD^+^ producing and consuming enzymes, suggests unique recognition features and dependency on the purine’s N7 moiety, which is likely essential for PARP1-mediated reactions.^67^

Yao Li discovered that DncV, a CDN synthetase from *V. cholerae*, can produce symmetrical and mixed c-di-GMP analogs using GTP, ^th^GTP, and ^tz^GTP (Figure 9).^68^ Due to the distinct conformation adopted by the cyclic products, placing the aromatic rings in close proximity, the enzymatic synthesis and both specific (rocR) and nonspecific (S1) phosphodiesterase-mediated degradation can be monitored in real time by fluorescence.^68^ Importantly, the emissive c-di-GMP analogs (e.g., c-G^th^GMP) induce type-I interferon production in eukaryotic cells, with some being more potent than c-di-GMP.^69^ While a range of activities have been observed and mechanistic insight into their cellular SAR is still lacking, the ability of the emissive surrogates to tune the innate immune response in eukaryotic cells is noteworthy.

## 7 A Word about Utility

**Figure 11:**
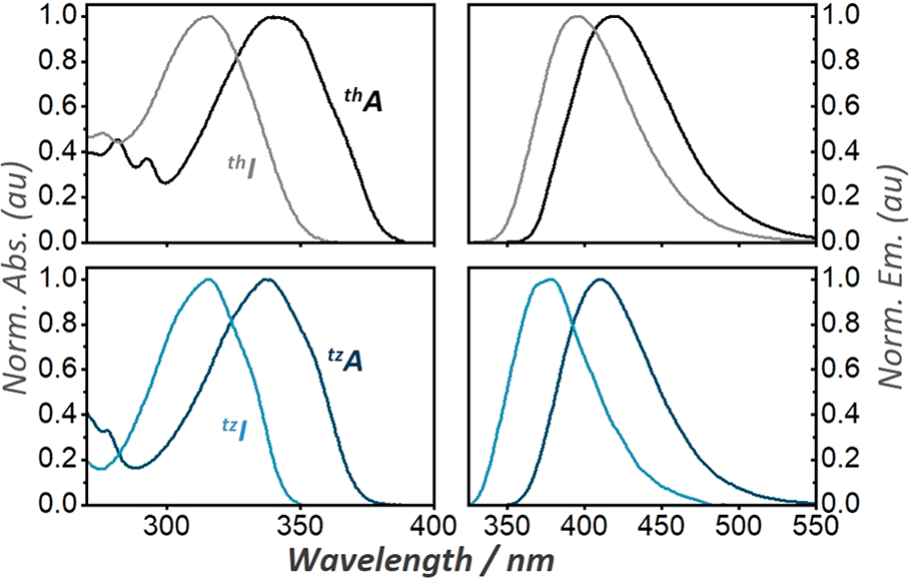
Normalized absorption (left) and emission (right) spectra of ^th^A (top, black) and ^tz^A (bottom, blue) as well as their corresponding deamination products ^th^I (gray) and ^tz^I (light blue), respectively, illustrating their distinct spectra and wavelength window.

While the intellectual gratification of developing new emissive nucleosides and learning about structure–photophysics relationships are immense, a tool has to ultimately serve a need and a purpose. Isomorphic fluorescent nucleosides possess significant advantages as well as certain shortcomings in their implementation in biological and biochemical assays. Fundamentally, as highly analogous surrogates, they are accommodated by various biochemical pathways and can frequently report in real time their binding and/or chemical transformations. Distinct absorption and emission open a spectral window, well separated from their natural counterparts, thus minimizing potential interference, which can be useful for operation in complex media and inhibitor discovery. Considering that numerous inhibitors of nucleoside-processing enzymes frequently contain the native chromophoric skeleton (e.g., EHNA, a common inhibitor of adenosine deaminase), spectroscopy-based tools (particularly absorption spectroscopy) might be subjected to substantial interference. Thus, shifting the operational spectral window by 60–100 nm away from the nonemissive canonical nucleobases and relying on visible emission is significant and highly beneficial for such applications (Figure 11).^57^

But is it all that matters? Certainly not. A simple caveat associated with the development and implementation of new chromophoric nucleosides is the synthetic effort required for their preparation. The biochemistry/biology community relies heavily on coupled assays and chimeric fluorescent protein expression. While such tools appear complex, they have been refined and “kitted”. In contrast, “elegant”, simplified assays based on isomorphic emissive nucleosides, which are not commercially available or easily accessible, face a hurdle and cannot be broadly implemented. This chicken and egg dilemma is difficult to untangle, but collaborations and sample sharing have increased the recognition of these nucleosides and their potential.

Similarly, due to 2-AP’s long history and its commercial dominance, a notion implying that “one emissive nucleoside fits all” might have mistakenly emerged. As mentioned, 2-AP’s high responsiveness, which has been exploited in numerous studies, is also its shortcoming. Briefly, 2AP undergoes significant sequence-dependent quenching in single- and double-stranded constructs.^54,55^ Despite an abundance of dark states, a residual emission may be detected. Such signals might be interpreted as reflecting the phenomenon under investigation while in reality emanating from minor but emissive populations with little or no biological relevance. Our first-generation emissive guanosine surrogate, ^th^G, does not suffer from such deficiencies and retains high emission levels in diverse contexts and can be reliably used in steady-state, time-resolved, and anisotropy measurements.^55^ It suffers, however, from other limitations, particularly the lack of the coordinating/basic nitrogen at the position corresponding to the purines’ N7, which could, in certain contexts, compromise functionality. This has largely been solved, albeit with about a 50% drop in the emission quantum yield, by introducing the highly isomorphic ^tz^G (Figure 6).^3^

Importantly, having the two emissive nucleoside families, ^th^N and ^tz^N, facilitates their use as mechanistic probes, assessing the involvement of N7 in biomolecular interactions by fluorescence. Furthermore, although more perturbing (and originally projected to be problematic), even the methylated thiopheno nucleosides have proven useful (see ^mth^C, Figure 8),^70^ assisting in demonstrating that certain enzymes can accommodate rather perturbing pyrimidine analogs. This has reaffirmed our notion that the performance of any probe, ideal or perturbing as it might be, is application- and implementation-dependent. Evaluating multiple distinct probes is frequently sensible and insightful.

In this context, it is important to appreciate that other motifs of emissive nucleosides certainly exist, and some have been put to excellent use as labels and sensors. Emissive 5-modified pyrimidines and 7-substituted-7-deazapurines, while perhaps viewed as perturbing, are frequently accepted by polymerases.^71^ This facilitates their incorporation into oligonucleotides and furthers their applications, such as studying protein–nucleic acid interactions.^71^ Emissive and responsive quinazolines have been developed and have shown promise for biophysically assessing folding processes.^40,72^ Other bright fluorophores, particularly tricyclic cytidine analogs (tC and derivatives), can be enzymatically incorporated into oligonucleotides and have been utilized for both in vitro biophysical and cellular imaging purposes.^73^ Such analogs tend to be brighter and less responsive than their isomorphic counterparts, which bodes well for imaging applications. For newcomers to the field, it is therefore critical to realize that the application and the sensitivity of the biological system studied to inevitable structural perturbations ultimately dictate a probe’s utility.

## 8 Discarded Motifs

One may wonder, after inspecting the structures shown above, whether related or isomeric structures have been explored. Not uncommonly, “negative” results, or less than optimal fluorophores, do not see the light of day. Indeed, over the past two decades, we have prepared and tested several alternative and related motifs. Frequently, mediocre photophysical features or compromised functionality, which could imply perturbing features or susceptibility to thermal or photochemical transformations, has resulted in abandoning such candidate fluorescent nucleosides.

**Figure 12:**
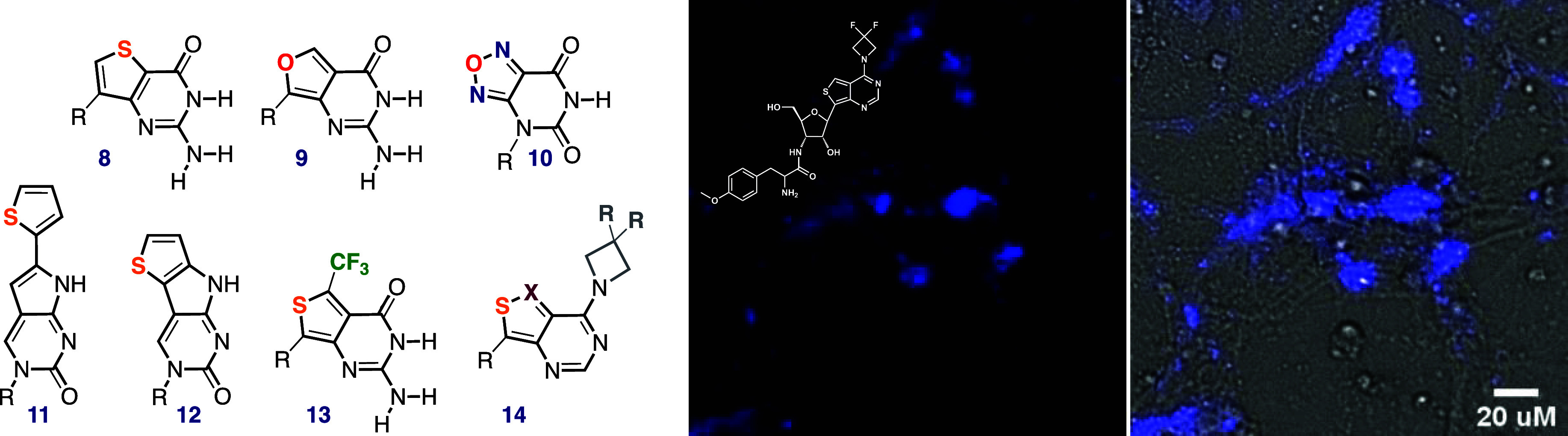
Previously “discarded” motifs (**8**–**12**) and recently developed emissive nucleosides (**13**, **14**). Direct imaging of an emissive puromycin analog (middle) and its overlay on a bright-field image (right).^4^

It is noteworthy that the isomeric thieno[3,2-*d*]pyrimidine core (**8**) could have served, in principle, as an alternative alphabet core (Figure 12). Although several derivatives have been prepared by David Jaramillo, preliminary photophysical evaluation had shown the U analogs to emit around 350 nm.^41^ This high-energy emission coupled with relatively low emission quantum yields (ϕ_F_ = 0.04–0.06) had steered us away from this motif. The furo[3,4-*d*]pyrimidine-2,4(1*H*,3*H*)-dione heterocyclic core (**9**) appears to be too reactive and undergoes undesired thermally induced transformations when incorporated into oligonucleotides as preliminarily observed by Daniel Palacios (Figure 12). While limited and not able to be expanded, the furazan analogue (**10**) was prepared early on by Renatus Sinkeldam but was also deemed inadequate due to its relatively poor emission intensity.

Motifs that are either synthetically challenging or cannot be expanded into related analogs have been studied but were left unutilized. Intriguing examples include the extended and fused thiopheno-pyrroloC analogs (**11** and **12**, respectively, Figure 12) prepared and studied by Mary Noé and Andro Ríos.^74^ While 10- to 20-fold brighter than the parent pyrroloC, the emission of both analogs was only slightly red-shifted to about 474 nm in water (vs 461 nm for pC). Their solvent-dependent emission illustrated the unpredictability and intricacies of photophysical features. Whereas the emission quantum yield of **11** is practically unresponsive to solvent polarity (ϕ_F_ = 0.43 and 0.47 in water and dioxane, respectively), **12** undergoes almost complete quenching in water (ϕ_F_ = 0.01 and 0.70 in water and dioxane, respectively).^74^

## 9 Summary, Prospects, Challenges, and Opportunities

Isomorphic and broadly functional emissive nucleosides have been designed, synthesized, and implemented. They have been shown to seamlessly replace their native counterparts in numerous contexts, providing a faithful optical window into biochemical transformations. Compromised (or elevated) performance has frequently shed light on subtle biological recognition features. Unlike bright, imaging-friendly fluorophores (e.g., cyanine dyes), such isomorphic emissive nucleosides normally display high photophysical sensitivity to their microenvironment. Although useful for in vitro applications, this may reflect a potential liability for cellular imaging. Additionally and perhaps ironically, another limitation of highly isomorphic nucleosides is that in vivo applications might be inherently compromised: a cell exposed to a nucleoside that is largely accommodated by diverse endogenous pathways might not withstand such an assault on its metabolic integrity.

Fluorophores with the footprint and photophysical characteristics of isomorphic nucleosides have rarely been explored for biological imaging, and their implementation represents a major technical challenge. Nonetheless, the potential utility of nonperturbing fluorescent nucleosides, nucleotides, and oligonucleotides in biophysics, chemical biology, biotechnology, drug discovery, and ultimately cell biology remains massive. In pursuing emissive nucleoside-based antibiotics, Kaivin Hadidi learned that replacing a naturally occurring Me_2_N group in thiopheno-puromycin analogs with substituted azetidines dramatically enhances emission quantum yields (e.g., **14**).^4,15^ Such inherently emissive analogs can be used to terminate ribosomal translation and label nascent peptides in live cells, which can be imaged by fluorescence microscopy without any follow-up labeling reactions (Figure 12).^4^ While this is our first foray into cellular imaging, it exemplifies that such methodologies are not beyond small responsive nucleosides. New analogs and progress made with multiphoton excitation may thus alleviate such perceived deficiencies and expand the horizons of such fascinating small fluorophores, particularly with the resurgence of nucleic acid research seen in recent years.

## Supporting Information Available

Fluorescence spectroscopy primer, summary of linear free energy relationship terms used, and supporting references (PDF)

## Abbreviations

ADA: adenosine deaminase
CDN: cyclic dinucleotide
MM: Michaelis–Menten
NAD: nicotinamide adenine dinucleotide
NTP: nucleoside triphosphate

## References

[ref1] Shin D.; Sinkeldam R. W.; Tor Y. Emissive RNA Alphabet. J. Am. Chem. Soc. 2011, 133, 14912–14915. 10.1021/ja206095a. 21866967 PMC3179766

[ref2] Sinkeldam R. W.; McCoy L. S.; Shin D.; Tor Y. Enzymatic Interconversion of Isomorphic Fluorescent Nucleosides: Adenosine Deaminase Transforms an Adenosine Analogue into an Inosine Analogue. Angew. Chem., Int. Ed. 2013, 52, 14026–14030. 10.1002/anie.201307064. PMC3947497 24288262

[ref3] Rovira A.; Fin A.; Tor Y. Chemical mutagenesis of an emissive RNA alphabet. J. Am. Chem. Soc. 2015, 137, 14602–14605. 10.1021/jacs.5b10420. 26523462 PMC5281058

[ref4] Hadidi K.; Steinbuch K. B.; Dozier L. E.; Patrick G. N.; Tor Y. Inherently Emissive Puromycin Analogues for Live Cell Labelling. Angew. Chem., Int. Ed. 2023, 62, e202216784 10.1002/anie.202216784. PMC10213139 36973168

[ref5] Rodriguez E. A.; Campbell R. E.; Lin J. Y.; Lin M. Z.; Miyawaki A.; Palmer A. E.; Shu X.; Zhang J.; Tsien R. Y. The Growing and Glowing Toolbox of Fluorescent and Photoactive Proteins. Trends Biochem. Sci. 2017, 42 (2), 111–129. 10.1016/j.tibs.2016.09.010. 27814948 PMC5272834

[ref6] Sinkeldam R. W.; Greco N. J.; Tor Y. Fluorescent Analogs of Biomolecular Building Blocks: Design, Properties and Applications. Chem. Rev. 2010, 110, 2579–2619. 10.1021/cr900301e. 20205430 PMC2868948

[ref7] Xu W.; Chan K. M.; Kool E. T. Fluorescent nucleobases as tools for studying DNA and RNA. Nat. Chem. 2017, 9, 1043–1055. 10.1038/nchem.2859. 29064490 PMC5819341

[ref8] aDaniels M.; Hauswirth W. Fluorescence of the purine and pyrimidine bases of the nucleic acids in neutral aqueous solution at 300 degrees K. Science 1971, 171, 675–677. 10.1126/science.171.3972.675. 5540307

[ref9] Gustavsson T.; Markovitsi D. Fundamentals of the Intrinsic DNA Fluorescence. Acc. Chem. Res. 2021, 54, 1226–1235. 10.1021/acs.accounts.0c00603. 33587613

[ref10] See Supporting Information for a fluorescence spectroscopy primer and for additional information on quantitative substituent and solvent effects.

[ref11] Klöcker N.; Weissenboeck F. P.; Rentmeister A. Covalent labeling of nucleic acids. Chem. Soc. Rev. 2020, 49, 8749–8773. 10.1039/D0CS00600A. 33084688 PMC7116832

[ref12] aSanborn M. E.; Connolly B. K.; Gurunathan K.; Levitus M. Fluorescence Properties and Photophysics of the Sulfoindocyanine Cy3 Linked Covalently to DNA. J. Phys. Chem. B 2007, 111, 11064–11074. 10.1021/jp072912u. 17718469

[ref13] Weber G. Excited States of Proteins. In Light and Life; McElroy W. D., Glass B., Eds.; Johns Hopkins Press: Baltimore, 1961; pp 82–106.

[ref14] aTeale F. W. J.; Weber G. Ultraviolet fluorescence of aromatic amino acids. Biochem. J. 1957, 65, 476–482. 10.1042/bj0650476. 13412650 PMC1199900

[ref15] Hadidi K.; Tor Y. Azetidines-Containing Fluorescent Purine Analogs: Synthesis and Photophysical Properties. Chem.—Eur. J. 2022, 28, e202200765 10.1002/chem.202200765. 35303392 PMC9133108

[ref16] Sinkeldam R. W.; Tor Y. To D or not to D? On estimating the microenvironment polarity of biomolecular cavities. Org. Biomol. Chem. 2007, 5, 2523–2528. 10.1039/b707719j. 18019524

[ref17] Ward D. C.; Reich E.; Stryer L. Fluorescence Studies of Nucleotides and Polynucleotides. 1. Formycin, 2-aminopurine riboside, 2,6-diaminopurine riboside and their derivatives. J. Biol. Chem. 1969, 244, 1228–1237. 10.1016/S0021-9258(18)91833-8. 5767305

[ref18] Dallmann A.; Dehmel L.; Peters T.; Mügge C.; Griesinger C.; Tuma J.; Ernsting N. P. 2-Aminopurine Incorporation Perturbs the Dynamics and Structure of DNA. Angew. Chem., Int. Ed. 2010, 49, 5989–5992. 10.1002/anie.201001312. 20632340

[ref19] Jones A. C.; Neely R. K. 2-aminopurine as a fluorescent probe of DNA conformation and the DNA–enzyme interface. Q. Rev. Biophys. 2015, 48, 244–279. 10.1017/S0033583514000158. 25881643

[ref20] Kirk S. R.; Luedtke N. W.; Tor Y. 2-Aminopurine as a Real-Time Probe of Enzymatic Cleavage and Inhibition of Hammerhead Ribozymes. Bioorg. Med. Chem. 2001, 9, 2295–2301. 10.1016/S0968-0896(01)00123-7. 11553468

[ref21] Secrist J. A.; Barrio J. R.; Leonard N. J.; Weber G. Fluorescent modifications of adenosine containing coenzymes. Biological activities and spectroscopic properties. Biochemistry 1972, 11, 3499–3506. 10.1021/bi00769a001. 4340904

[ref22] Leonard N. J.; Barrio J. R. Etheno-Substituted Nucleotides and Coenzymes: Fluorescence and Biological Activity. Crit. Rev. Biochem. 1984, 15, 125–199. 10.3109/10409238409102299. 6365449

[ref23] Otter B. A.; Patil S. A.; Klein R. S.; Ealick S. E. A Corrected Structure for Pyrrolosine. J. Am. Chem. Soc. 1992, 114, 668–671. 10.1021/ja00028a038.

[ref24] Greco N. J.; Tor Y. Simple Fluorescent Pyrimidine Analogs Detect the Presence of DNA Abasic Sites. J. Am. Chem. Soc. 2005, 127, 10784–10785. 10.1021/ja052000a. 16076156

[ref25] Greco N. J.; Tor Y. Furan Decorated Nucleoside Analogues as Fluorescent Probes: synthesis, photophysical evaluation and site-specific incorporation. Tetrahedron 2007, 63, 3515–1527. 10.1016/j.tet.2007.01.073. 18431439 PMC1868554

[ref26] Sinkeldam R. W.; Wheat A.; Boyaci H.; Tor Y. Emissive Nucleosides as Molecular Rotors. ChemPhysChem. 2011, 12, 567–570. 10.1002/cphc.201001002. 21344595 PMC3102795

[ref27] aSrivatsan S. G.; Tor Y. Fluorescent Pyrimidine Ribonucleotide: Synthesis, Enzymatic Incorporation, and Utilization. J. Am. Chem. Soc. 2007, 129, 2044–2053. 10.1021/ja066455r. 17256858 PMC2517582

[ref28] Greco N. J.; Sinkeldam R. W.; Tor Y. An emissive C analog distinguishes between G, 8-oxoG and T. Org. Lett. 2009, 11, 1115–1118. 10.1021/ol802656n. 19196162 PMC2765557

[ref29] aNuthanakanti A.; Boerneke M. A.; Hermann T.; Srivatsan S. G. Structure of the Ribosomal RNA Decoding Site Containing a Selenium-Modified Responsive Fluorescent Ribonucleoside Probe. Angew. Chem., Int. Ed. Engl. 2017, 56 (10), 2640–2644. 10.1002/anie.201611700. 28156044 PMC5397316

[ref30] aCservenyi T. Z.; Van Riesen A. J.; Berger F. D.; Desoky A.; Manderville R. A. A Simple Molecular Rotor for Defining Nucleoside Environment within a DNA Aptamer–Protein Complex. ACS Chem. Biol. 2016, 11, 2576–2582. 10.1021/acschembio.6b00437. 27447371

[ref31] Sinkeldam R. W.; Greco N.; Tor Y. Polarity of major grooves explored by using an isosteric emissive nucleoside. ChemBioChem. 2008, 9, 706–709. 10.1002/cbic.200700714. 18286575

[ref32] Sinkeldam R. W.; Hopkins P. A.; Tor Y. Modified 6-Aza Uridines: Highly Emissive pH Sensitive Fluorescent Nucleosides. ChemPhysChem 2012, 13, 3350–3356. 10.1002/cphc.201200375. 22777983 PMC3466602

[ref33] Hopkins P. A.; Sinkeldam R. W.; Tor Y. Visibly Emissive and Responsive Extended 6-Aza-Uridines. Org. Lett. 2014, 16, 5290–5293. 10.1021/ol502435d. 25285451 PMC4201329

[ref34] Lane R. S. K.; Jones R.; Sinkeldam R. W.; Tor Y.; Magennis S. W. Two-Photon-Induced Fluorescence of Isomorphic Nucleobase Analogs. ChemPhysChem. 2014, 15, 867–871. 10.1002/cphc.201400031. 24604669 PMC4041282

[ref35] Nobis D.; Fisher R. S.; Simmermacher M.; Hopkins P. A.; Tor Y.; Jones A. C.; Magennis S. W. Single-Molecule Detection of a Fluorescent Nucleobase Analogue via Multiphoton Excitation. J. Phys. Chem. Lett. 2019, 10, 5008–5012. 10.1021/acs.jpclett.9b02108. 31397575 PMC7024020

[ref36] Xie Y.; Dix A. V.; Tor Y. FRET Enabled Real Time Detection of RNA–Small Molecule Binding. J. Am. Chem. Soc. 2009, 131, 17605–17614. 10.1021/ja905767g. 19908830 PMC3031783

[ref37] Xie Y.; Dix A. V.; Tor Y. Antibiotic Selectivity for Prokaryotic vs. Eukaryotic Decoding Sites. Chem. Commun. 2010, 46, 5542–5544. 10.1039/c0cc00423e. PMC3035430 20464029

[ref38] Xie Y.; Maxson T.; Tor Y. Fluorescent ribonucleoside as a FRET acceptor for tryptophan in native proteins. J. Am. Chem. Soc. 2010, 132, 11896–11897. 10.1021/ja105244t. 20690779 PMC2941768

[ref39] Baker C. M.; Grant H. G. Role of Aromatic Amino Acids in Protein–Nucleic Acid Recognition. Biopolymers 2007, 85, 456–470. 10.1002/bip.20682. 17219397

[ref40] Other emissive quinazolines: Mata G.; Luedtke N. W. Synthesis and Solvatochromic Fluorescence of Biaryl Pyrimidine Nucleosides. Org. Lett. 2013, 15, 2462–2465. 10.1021/ol400930s. 23656574

[ref41] Tor Y.; Del Valle S.; Jaramillo D.; Srivatsan S. G.; Rios A.; Weizman H. Designing new isomorphic fluorescent nucleobase analogues: the thieno[3,2-*d*]pyrimidine core. Tetrahedron 2007, 63, 3608–3614. 10.1016/j.tet.2007.01.075.

[ref42] McCoy L. S.; Shin D.; Tor Y. Isomorphic emissive GTP surrogate facilitates initiation and elongation of in vitro transcription reactions. J. Am. Chem. Soc. 2014, 136, 15176–15184. 10.1021/ja5039227. 25255464 PMC4227834

[ref43] Li Y.; Fin A.; McCoy L.; Tor Y. Polymerase-mediated site-specific incorporation of a synthetic fluorescent isomorphic G surrogate into RNA. Angew. Chem., Int. Ed. 2017, 56, 1303–1307. 10.1002/anie.201609327. PMC5241218 28000329

[ref44] Lyon S.; Gopalan V. A T7 RNA Polymerase Mutant Enhances the Yield of 5′-Thienoguanosine-Initiated RNAs. ChemBioChem. 2018, 19, 142–146. 10.1002/cbic.201700538. 29115013 PMC6047071

[ref45] aSamanta P. K.; Manna A. K.; Pati S. K. Thieno Analogues of RNA Nucleosides: A Detailed Theoretical Study. J. Phys. Chem. B 2012, 116, 7618–7626. 10.1021/jp301752k. 22671305

[ref46] Srivatsan S. G.; Greco N. J.; Tor Y. A highly emissive fluorescent nucleoside that signals the activity of toxic ribosome-inactivating proteins. Angew. Chem., Int. Ed. 2008, 47, 6661–6665. 10.1002/anie.200802199. PMC2633406 18683267

[ref47] Shin D.; Lönn P.; Dowdy S. F.; Tor Y. Cellular activity of siRNA oligonucleotides containing synthetic isomorphic nucleoside surrogates. Chem. Commun. 2015, 51, 1662–1665. 10.1039/C4CC08809C. PMC4298453 25500944

[ref48] aPark S.; Otomo H.; Zheng L.; Sugiyama H. Highly emissive deoxyguanosine analogue capable of direct visualization of B–Z transition. Chem. Commun. 2014, 50, 1573–1575. 10.1039/c3cc48297a. 24382561

[ref49] Otomo H.; Park S.; Yamamoto S.; Sugiyama H. Amplification of fluorescent DNA through enzymatic incorporation of a highly emissive deoxyguanosine analogue. RSC Adv. 2014, 4, 31341–31344. 10.1039/C4RA05678G.

[ref50] Kilin V.; Barthes N.; Michel B.; Boudier C.; Yashchuk V.; Mousli M.; Ruff M.; Granger F.; Bronner C.; Tor Y.; Burger A.; Mély Y. Dynamics of methylated cytosine flipping by UHRF1. J. Am. Chem. Soc. 2017, 139, 2520–2528. 10.1021/jacs.7b00154. 28112929 PMC5335914

[ref51] Liu W.; Shin D.; Tor Y.; Cooperman B. S. Monitoring translation with modified mRNAs strategically labeled with isomorphic fluorescent guanosine mimetics. ACS Chem. Biol. 2013, 8, 2017–2023. 10.1021/cb400256h. 23865809 PMC3783585

[ref52] Liu W.; Shin D.; Ng M.; Sanbonmatsu K.; Tor Y.; Cooperman B. S. Stringent nucleotide recognition by the ribosome at the middle codon position. Molecules 2017, 22, 1427 10.3390/molecules22091427. 28850078 PMC5753802

[ref53] Han J. H.; Park S.; Hashiya F.; Sugiyama H. Approach to the Investigation of Nucleosome Structure by Using the Highly Emissive Nucleobase ^th^dG–tC FRET Pair. Chem.—Eur. J. 2018, 24, 17091–17095. 10.1002/chem.201803382. 30207401

[ref54] Sholokh M.; Sharma R.; Shin D.; Das R.; Zaporozhets O.; Tor Y.; Mély Y. Conquering 2-Aminopurine’s Deficiencies: Highly Emissive Isomorphic Guanosine Surrogate Faithfully Monitors Guanosine Conformation and Dynamics in DNA. J. Am. Chem. Soc. 2015, 137, 3185–3188. 10.1021/ja513107r. 25714036 PMC4357565

[ref55] aKuchlyan J.; Martinez-Fernandez L.; Mattia M.; Gavvala K.; Ludovic R.; Didier P.; Tor Y.; Improta R.; Mély Y. What makes thienoguanosine an outstanding fluorescent DNA probe?. J. Am. Chem. Soc. 2020, 142, 16999–17014. 10.1021/jacs.0c06165. 32915558 PMC7544670

[ref56] Rovira A. R.; Fin A.; Tor Y. Expanding a fluorescent RNA Alphabet: synthesis, photophysics and utility of isothiazole-derived purine nucleoside surrogates. Chem. Sci. 2017, 8, 2983–2993. 10.1039/C6SC05354H. 28451365 PMC5380116

[ref57] Ludford P. T. III; Rovira A. R.; Fin A.; Tor Y. Fluorescing Isofunctional Ribonucleosides: Assessing Adenosine Deaminase Activity and Inhibition. ChemBioChem. 2019, 20, 718–726. 10.1002/cbic.201800665. 30566279 PMC6467514

[ref58] Adamek R. N.; Ludford P.; Duggan S. M.; Tor Y.; Cohen S. M. Identification of Adenosine Deaminase Inhibitors by Metal-binding Pharmacophore Screening. ChemMedChem. 2020, 15, 2151–2156. 10.1002/cmdc.202000271. 32729197 PMC7815202

[ref59] Mizrahi R. A.; Shin D.; Sinkeldam R. W.; Phelps K. J.; Fin A.; Tantillo D. J.; Tor Y.; Beal P. A. Fluorescent Adenosine Analog as a Substrate for an A-to-I RNA Editing Enzyme. Angew. Chem., Int. Ed. 2015, 54, 8713–8716. 10.1002/anie.201502070. PMC4532316 26095193

[ref60] Pokharel S.; Jayalath P.; Maydanovych O.; Goodman R. A.; Wang S. C.; Tantillo D. J.; Beal P. A. Matching Active Site and Substrate Structures for an RNA Editing Reaction. J. Am. Chem. Soc. 2009, 131, 11882–11891. 10.1021/ja9034076. 19642681

[ref61] Bucardo M. S.; Wu Y.; Ludford P. T. III; Li Y.; Fin A.; Tor Y. Real-Time Monitoring of Human Guanine Deaminase Activity by an Emissive Guanine Analog. ACS Chem. Biol. 2021, 16, 1208–1214. 10.1021/acschembio.1c00232. 34190533 PMC9109600

[ref62] Ludford P. T. III; Li Y.; Yang S.; Tor Y. Cytidine deaminase can deaminate fused pyrimidine ribonucleosides. Org. Biomol. Chem. 2021, 19, 6237–6243. 10.1039/D1OB00705J. 34019616 PMC8295196

[ref63] Wierzchowski J.; Antosiewicz J. M.; Shugar D. 8-Azapurines as isosteric purine fluorescent probes for nucleic acid and enzymatic research. Mol. BioSyst. 2014, 10, 2756–2774. 10.1039/C4MB00233D. 25124808

[ref64] Vranken C.; Fin A.; Tufar P.; Hofkens J.; Burkart M. D.; Tor Y. Chemoenzymatic synthesis and utilization of a SAM analog with an isomorphic nucleobase. Org. Biomol. Chem. 2016, 14, 6189–6192. 10.1039/C6OB00844E. 27270873 PMC4927405

[ref65] Rovira A. R.; Fin A.; Tor Y. Emissive synthetic cofactors: An isomorphic, isofunctional and responsive NAD+ analogue. J. Am. Chem. Soc. 2017, 139, 15556–15559. 10.1021/jacs.7b05852. 29043790 PMC5766281

[ref66] Halle F.; Fin A.; Rovira A. R.; Tor Y. Emissive synthetic cofactors: enzymatic interconversions of tzA analogues of ATP, NAD+, NADH, NADP+ and NADPH. Angew. Chem., Int. Ed. 2018, 57, 1087–1090. 10.1002/anie.201711935. PMC5771816 29228460

[ref67] Feldmann J.; Li Y.; Tor Y. Emissive Synthetic Cofactors: A Highly Responsive NAD+ Analogue Reveals Biomolecular Recognition Features. Chem.—Eur. J. 2019, 25, 4379–4389. 10.1002/chem.201805520. 30648291 PMC6944523

[ref68] Li Y.; Ludford P. T. III; Fin A.; Rovira A.; Tor Y. Enzymatic Synthesis and Applications of Fluorescent Cyclic Dinucleotides. Chem.—Eur. J. 2020, 26, 6076–6084. 10.1002/chem.202001194. 32157755 PMC7220823

[ref69] Li Y.; Fin A.; Rovira A. R.; Su Y.; Dippel A. B.; Valderrama J. A.; Riestra A.; Nizet V.; Hammond M. C.; Tor Y. Tuning the Innate Immune Response to Cyclic Dinucleotides Using Atomic Mutagenesis. ChemBioChem. 2020, 21, 2595–2598. 10.1002/cbic.202000162. 32346955 PMC7494572

[ref70] Ludford P. T.; Yang S.; Tor Y. A New Variant of Emissive RNA Alphabets. Chem.—Eur. J. 2022, 28, e202104472 10.1002/chem.202104472. 35018663 PMC8891053

[ref71] aSeela F.; Zulauf M.; Sauer M.; Deimel M. 7-Substituted 7-Deaza-29-deoxyadenosines and 8-Aza-7-deaza-29-deoxyadenosines: Fluorescence of DNA-Base Analogues Induced by the 7-Alkynyl Side Chain. Helv. Chim. Acta 2000, 83, 910–927. 10.1002/(SICI)1522-2675(20000510)83:5<910::AID-HLCA910>3.0.CO;2-4.

[ref72] Karimi A.; Börner R.; Mata G.; Luedtke N. W. A Highly Fluorescent Nucleobase Molecular Rotor. J. Am. Chem. Soc. 2020, 142, 14422–14426. 10.1021/jacs.0c05180. 32786749

[ref73] aWilhelmsson L. M. Fluorescent nucleic acid base analogues. Q. Rev. Biophys. 2010, 43, 159–183. 10.1017/S0033583510000090. 20478079

[ref74] Noé M. S.; Ríos A. C.; Tor Y. Design, Synthesis and Spectroscopic Properties of Extended and Fused Pyrrolo-dC and Pyrrolo-C Analogs. Org. Lett. 2012, 14, 3150–3153. 10.1021/ol3012327. 22646728 PMC3426657

