# Supplementary material for: Isomorphic Fluorescent Nucleosides
Source: Acc Chem Res. 2024 Apr 13;57(9):1325–35. doi: 10.1021/acs.accounts.4c00042 (PMC11079976; doi:10.1021/acs.accounts.4c00042)
Supplement: Supplementary file 1 — ar4c00042_si_001.pdf [file ar4c00042_si_001.pdf]

Supporting Information
for

Isomorphic Fluorescent Nucleosides

Yitzhak Tor*

Department of Chemistry and Biochemistry, University of California, San Diego, 9500 Gilman Drive, La Jolla, CA 92093, United States

This Supporting Information includes a fluorescence spectroscopy primer, a summary of Linear Free Energy Relationships terms used, and supporting references

A fluorescence spectroscopy primer

Figure S1 shows a basic Jablonski diagram. Excitation of a chromophore rapidly generates the Franck-Condon state (within about 10^{-15} s). The efficiency of this electronic reorganization, from the ground to excited state(s), corresponds to the chromophore's absorption cross-section (σ), which is proportional to its extinction coefficient (ε). Extinction coefficients are typically listed for the peak, or maximum absorption wavelength (λ_{max}), thus providing a measure of how capable a chromophore is in absorbing a photon of a given wavelength/energy.

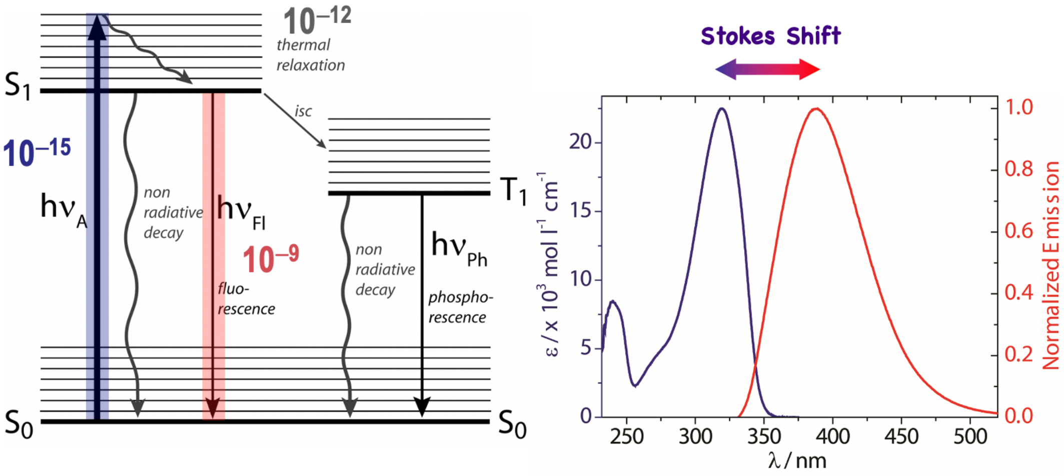

Figure S1. Left: a basic Jablonski diagram, illustrating key electronic transitions (absorption in blue and emission in ref) and the time scale associated with the excitation and relaxation processes. Right: a typical solution absorption spectrum of a chromophore (blue) and the associated normalized emission band (red). The energetic difference between the absorption and emission maxima is referred to as the ***Stokes shift***.

Vibrational relaxation (within 10^{-12}–10^{-10} s) rapidly populates the lowest vibronic state of the chromophore's excited state (Figure S1). This relaxation process, which, as shown, populates a lower energy emissive state, thus accounts for the lower emission energy (longer wavelength) of a chromophore compared with its higher excitation energy (shorter wavelength). The energetic difference between the absorption and the emission maxima is commonly referred to as the chromophore's Stokes shift. To calculate this value, it is advisable to convert the measured absorption and emission wavelength peaks into energy units, such as cm^{-1}, and subtract the two values. This is typically more meaningful than reporting the difference in wavelength (nm), as it accounts for the actual energetic difference.

Typical organic chromophores, such as the ones discussed in this *Accounts of Chemical Research* article, reside in their excited state for a period of (0.5–20) × 10^{-9} s. Both radiative and nonradiative processes can facilitate the decay back to the ground state, and the excited state lifetime reflects the sum of the various relaxation processes (τ_0). The fraction responsible for emitting a photon, or the fluorescence lifetime (τ), relates to the emission quantum yield of the chromophores ($\Phi = \tau/\tau_0$). In simplified terms, the quantum yield can be viewed as the number of photons emitted per number of photons absorbed.

For certain studies, particularly fluorescence imaging, the brightness (εΦ) of a fluorophore is reported, which is the product of the molar absorptivity (ε) and the fluorescence quantum yield (Φ). This could be useful when comparing the utility of two fluorophores with similar fluorescence quantum yields but distinct molar absorptivities.

Useful resources include:

1) Lakowicz, J. R. *Principles of Fluorescence Spectroscopy*, 3rd ed, Springer: New York, 2006
2) Sinkeldam, R. W.; Greco, N. J.; Tor, Y. Fluorescent Analogs of Biomolecular Building Blocks: Design, Properties and Applications. *Chem. Rev.* **2010***, 110,* 2579–2619.

Correlating photophysical features

When systematically studying the effect of structural modifications (or different substituents) it is useful to correlate experimental data with established quantitative empirical scales.

Substituent parameters

The **Hammett substituent constants** (or parameters) reflect the electronic features of a substituent (e.g., electron donating or withdrawing). They were derived from the Hammett equation, which is a linear free-energy relationship (LFER), originally used to the evaluate the effect of substituents on the equilibrium dissociation constants of benzoic acids ($\log K/K_o = \sigma$, where K is the dissociation constant obtained for a substituted benzoic acid and K_o is the dissociation constant of benzoic acid). For example, electron withdrawing groups (e.g., nitro) facilitate dissociation and have positive σ values, while electron donating groups (e.g., dimethylamino) have negative σ values. Numerous scales have since been developed, reflecting the substituent position (σ_p or σ_m), contributions from resonance stabilization, etc. See reference 1.

Solvent polarity

Reichardt's $E_T(30)$ is an empirical, microscopic solvent polarity scale, which is based on Reichardt's dye, a highly solvatochromic zwitterionic chromophore (see Figure 2 below and references 2 and 3). The dye is dissolved in the solvent of choice, the absorption spectrum is taken, and the maximum absorption wavelength (λ_{max}) is extracted. The $E_T(30)$ value (in kcal/mol) is obtained as follows:

$$E_T(30) = 28591/\lambda_{max}$$

A simplified energy diagram (Figure 2) reflects the impact of solvent polarity on the ground/excited state energies and hence on the absorption maximum of the dissolved dye. As a zwitterionic molecule (Figure 2), higher polarity stabilizes the dye's ground state. Since vertical excitation (Franck-Condon principle) is an extremely fast process (Figure 1), higher polarity "destabilizes" the Franck-Condon state, as the solvation sphere remains organized in its ground state ("more polar") configuration, leading to a higher energy for its excited state (hence higher absorption energy = shorter wavelength).

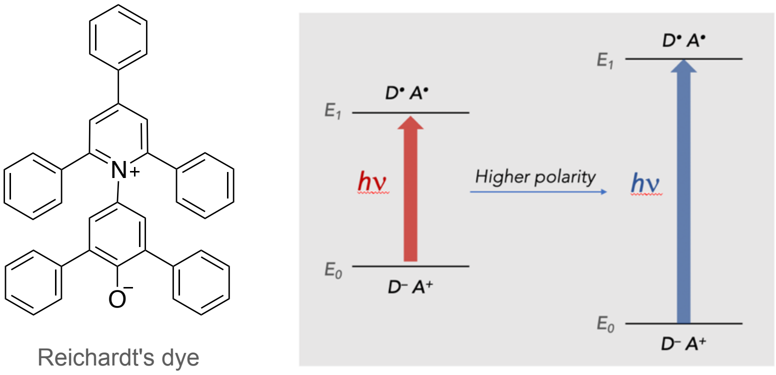

Figure S2. *Left*: Reichardt's zwitterionic dye is the chromophore used to determine the $E_T(30)$ values of a solvents/solvent mixtures. *Right*: Simplified photophysical considerations associated with ground–excited state energies as solvent polarity changes from less to more polar.

For photophysical studies we have always recommended using microscopic solvent polarity scales, such as the Reichardt's $E_T(30)$ scale, over the use of dielectric constants, which reflect a bulk solvent property (see reference 4 below).

<u>For additional reading, see:</u>

1) Anslyn, E. V.; Dougherty, D. A. *Modern Physical Organic Chemistry*. University Science Books, 2006.
2) Reichardt, C. *Solvents and solvent effects in organic chemistry*, VCH, Weinheim, 1988.
3) Reichardt, C. Solvatochromic Dyes as Solvent Polarity Indicators. *Chem. Rev.*, **1994**, *94*, 2319– 2358.
4) Sinkeldam, R. W.; Tor, Y. To D or not to D? On estimating the microenvironment polarity of biomolecular cavities. *Org. Biomol. Chem.* **2007**, *5*, 2523–2528.
